# Associations Between Self-Esteem With Diverse Deleterious Mental Health Outcomes Among Older Adults

**DOI:** 10.1093/geroni/igaa057.974

**Published:** 2020-12-16

**Authors:** Marissa Pifer, Daniel Segal, Meghan Marty

**Affiliations:** University of Colorado at Colorado Springs, Colorado Springs, Colorado, United States

## Abstract

Research suggests meaningful relationships between self-esteem and various deleterious mental health outcomes. However, these relationships are less well understood in older adults, where age-specific models of self-esteem are lacking. In this study, older adult participants (N = 284, M age = 73.3 years) anonymously completed a series of questionnaires as part of a larger battery. Measures examined for this study included the Single-Item Self-Esteem Scale (SISE), Patient Health Questionnaire (PHQ-9), Beck Hopelessness Scale (BHS), Three-Item Loneliness Scale (3LS), Sense of Belonging Inventory – Psychological Subscale (SOBI-P), and the Geriatric Anxiety Scale (GAS). A multiple regression was conducted to examine how these mental health variables predict self-esteem. The model explained a significant 39% of the variance in self-esteem, F (5,234) = 29.68, p < .001, with two significant predictors. Specifically, lower scores on the PHQ-9 (β = -3.06, p < .01) predicted higher scores on the SISE, whereas higher scores SOBI-P (β = .27, p < .01) predicted higher scores on the SISE. Scores on the 3LS (β = -.09), BHS (β = -.03), and GAS (β = -.03) did not emerge as significant predictors in the model. Results from this study indicate that lower depression and higher sense of belonging have unique contributions to self-esteem for older adults, controlling for the shared variance from other mental health variables, such as loneliness, hopelessness, and anxiety. Although causality was not assessed, future prospective studies should examine whether interventions focusing on reducing depression or increasing feelings of belongingness enhance self-esteem for older individuals.

